# Small tube-nosed seabirds fledge on the full moon and throughout the lunar cycle

**DOI:** 10.1098/rsbl.2023.0290

**Published:** 2023-12-06

**Authors:** Sydney M. Collins, April Hedd, William A. Montevecchi, Tori V. Burt, David R. Wilson, David A. Fifield

**Affiliations:** ^1^ Cognitive and Behavioural Ecology Program, Department of Biology, Memorial University of Newfoundland and Labrador, St John's, Canada; ^2^ Department of Psychology, Memorial University of Newfoundland and Labrador, St John's, Canada; ^3^ Wildlife Research Division, Environment and Climate Change Canada, Mount Pearl, Newfoundland and Labrador, Canada

**Keywords:** anthropogenic light, *Hydrobates leucorhous*, lunar phase, seabird, passive integrated transponder, radio-frequency identification

## Abstract

Many seabirds are attracted to anthropogenic light, and the risk is greater for recent fledglings. Moon phase predicts the probability of stranding (fewer birds strand on the full moon), but it remains uncertain whether moon phase is associated with when young seabirds fledge. Fledging behaviour of nocturnal, burrowing seabirds can be difficult to monitor using traditional methods but can provide insight into environmental factors that influence the risk of stranding. We used passive integrated transponder tags to monitor the fledging dates and times of Leach's storm-petrel (*Hydrobates leucorhous*) chicks across four breeding seasons (2017, 2018, 2021, 2022) at a major colony in Newfoundland and Labrador, Canada. We also assessed whether moon phase and incident illumination related to fledging date and time. The median fledge time was 1.6 h after sunset (0.6–11.7 h). The median fledge date was 10 October, and fledging dates ranged from 13 September to 13 November. Most importantly, moon phase was not associated with the time and date that Leach's storm-petrel chicks fledged. These results suggest that recently fledged storm-petrels are less attracted to anthropogenic light during high levels of natural illumination, which could indicate periods of higher stranding risk and help concentrate conservation efforts.

## 1. Introduction

Seabirds are one of the most at-risk groups of birds, and attraction to anthropogenic light is a risk for at least 73 seabird species, mainly procellariiforms [1–4]. Globally, thousands of seabirds strand annually around brightly lit coastal and offshore structures [2–7]. Stranded seabirds are subject to predation, dehydration, starvation, collisions with structures or vehicles, and oiling or injury by machinery [2]. Most seabirds that strand around anthropogenic light sources are recent fledglings and juveniles [5–8], which is evident during episodic mass stranding events involving hundreds to thousands of birds stranding within hours or days at a single site [6].

Moon phase has been considered to influence stranding [5]. Previous studies have observed that procellariiforms tend to strand the night they fledge [9,10], and that fewer tend to strand on nights with a full moon [5–8,11]. Further, adults tend to be less active at the colony during the full moon [12–16]. Together, these results suggest that nocturnal seabirds avoid fledging on nights when the moon is fuller [17], yet few studies have assessed this hypothesis [15,18,19].

In the North Atlantic, Leach's storm-petrels (*Hydrobates leucorhous*) are the most nocturnally active burrowing seabird species and the most abundant seabird species found stranded near anthropogenic light [5,7]. Ascertaining the factors that predict fledging of Leach's storm-petrels could help predict stranding events, but monitoring their fledging behaviour is difficult. First, storm-petrels are nocturnal at colonies [20], so our ability to observe the time and date of fledging is limited. Second, chicks may leave the burrow for several hours or days before returning [20], so an empty burrow does not necessarily indicate that the chick fledged.

To circumvent these challenges, we used passive integrated transponder (PIT) tags to remotely monitor fledging dates and times of Leach's storm-petrel chicks. Our specific objectives were to determine (1) the peak and range of fledging date and time and (2) whether fledging is associated with moon phase and illumination. We predicted that, relative to moon conditions available throughout the fledging period, proportionally fewer storm-petrel chicks fledge (1) on nights closer to the full moon, and (2) at times of night when incident light from the moon is greater [17]. Knowledge of fledging time and any coordination with environmental factors will enhance our ability to predict mass-stranding events and allow more concentrated monitoring during the periods of highest risk.

## 2. Methods

### (a) Field methods

#### (i) Field site

We studied Leach's storm-petrel chicks on Gull Island (47.26265, −52.77187), Witless Bay, Newfoundland and Labrador, Canada from 2017 to 2022. Gull Island supports approximately 180 000 breeding pairs of Leach's storm-petrels [21]. Chicks were monitored across six plots distributed along the southwestern side of the island (electronic supplementary material, figure S1).

#### (ii) Passive integrated transponder tag setup

Cylindrical glass 150 kHz PIT tags were set inside a custom three-dimesional printed leg band (either 12 × 2.12 mm CoreRFID model SOK027, 0.25 g total weight, or 10 × 2.12 mm Cyntag model 601205–248, 0.15 g total weight) and mounted on the leg of Leach's storm-petrel chicks (electronic supplementary material, figure S2*a*). Each chick was banded with a unique stainless steel identification band on the other leg, weighed and measured for wing chord length. Chick banding began in late August or early September of each year. Some chicks (less than 10%) may have fledged before banding occurred. Leach's storm-petrels have high hatching asynchrony [20], so not all chicks that were banded were large enough to be equipped with a PIT tag. These chicks were revisited later in the season when possible or were not included in this study. Tag reader antennae (electronic supplementary material, figure S2*b*) consisted of wire coils wrapped around custom three-dimensional-printed plastic cylinders (72 mm diameter by 20 mm deep) and a tuning capacitor. The antennae were inserted into the mouth of the burrow and secured in the ground using garden stakes. Each antenna was connected to a custom-made circuit board housed inside a Pelican case, which recorded the time and identification of the bird as it passed through the antenna. Video footage indicates that the antennae did not impede the storm-petrels' movement into or out of the burrow. The circuit board recorded system information (e.g. antenna frequency, battery voltage, etc.) and re-tuned the antenna every 30 min, which allowed us to identify the occurrence of system failures.

### (b) Verification of fledging

The final read at the burrow for each chick was considered the time and date of fledging. We could not physically verify fledging because (1) dead chicks sometimes become buried in the burrow chamber and cannot be detected by researchers during burrow inspections (pers. observation), (2) chicks may die outside the burrow while exploring [20], and (3) researcher access to the colony can be limited during the fledging period due to inclement weather. We, therefore, estimated the age of each chick at banding to determine whether the chick was old enough to fledge by the date of the last read. We estimated chick age from wing length from an equation derived by R. A. Mauck (unpubl.) using known-aged chicks at Kent Island, New Brunswick. A chick was assumed to have fledged if its estimated age at last read exceeded 56 days, as this represents the minimum fledging age observed across multiple colonies [20].

### (c) Statistical methods

All analyses were conducted using R v. 4.2.2 [22]. Summary statistics were calculated for fledging dates and times (Fledging data and code: Dryad (doi:10.5061/dryad.2bvq83bws [23]). ANOVAs were used to determine differences among years in fledging date and time. Kruskall–Wallace tests were used when data were non-normal.

Average illuminated per cent of the moon (AIPM, representing moon phase) on the night of fledging and an incident moon illumination index (IMII) at the time of fledging (i.e. the final read at the burrow) were calculated by the package *moonlit* (see electronic supplementary material for details) [24]. AIPM at peak fledge date was plotted to assess consistency among years. Kolmogorov–Smirnov tests examined whether the distribution of AIPM on fledging night and IMII at the time of fledging differed from the distribution of AIPM or IMII, respectively, throughout the fledging season across years (see electronic supplementary material for details). One-proportion z-tests at 5% AIPM or IMII intervals examined differences in observed versus expected fledgling proportions for the Kolmogorov–Smirnov tests. A chi-squared test examined whether chicks were more likely to fledge when the moon was below the horizon depending on AIPM categorized into quarters. Supplementary analyses regarding associations between cloud cover [25,26] and fledging date, and age at fledging and moon conditions, are in the electronic supplementary material (table S1, figures S3 and S4).

## 3. Results

**Figure 1.**
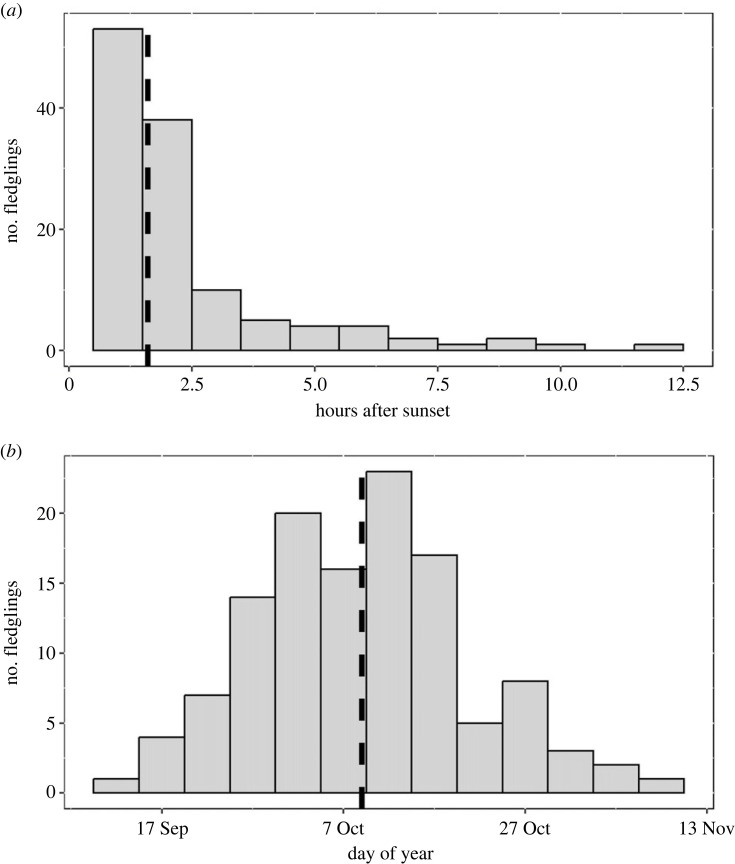
Histograms and median (black dashed line) (*a*) time after sunset and (*b*) day of year that Leach's storm-petrel chicks fledged from Gull Island, Witless Bay, Newfoundland and Labrador, Canada across 4 study years (*n* = 121 chicks).

In 2017, 2018, 2021 and 2022, 123 chicks were tracked using PIT tag technology (table 1). Based on the estimated chick age at fledging, two chicks were deemed too young to fledge at the time of their final read and were eliminated from the sample (final sample *n* = 121). The median fledge date of all chicks was 10 October (IQR: 15.0 days, range: 13 September to 13 November), and the median fledge time was 1.6 h after sunset (IQR: 1.3, range: 0.6–11.7 h) (table 1, figure 1, electronic supplementary material, S5).

**Table 1.** Summary statistics of the fledge date and time ± IQR (range) of Leach's storm-petrel chicks from Gull Island, Witless Bay, Newfoundland and Labrador, Canada. All times are in Newfoundland Daylight Time (NDT).

year	sample size	median date (days)	median time (hours)	median time past sunset (hours)
2017	30	11 Oct ± 14.3 days (19 Sept–29 Oct)	19.55 ± 1.3 h (19.17–05.30)	1.4 ± 1.3 h (0.7–11.7)
2018	42	07 Oct ± 16.8 days (19 Sept–31 Oct)	20.17 ± 0.9 h (18.47–03.57)	1.6 ± 0.9 h (0.9–9.3)
2021	9	28 Sept ± 13.0 days (25 Sept–18 Oct)	20.20 ± 1.1 h (19.11–01.11)	1.5 ± 1.2 h (1.0–6.9)
2022	40	11 Oct ± 18.8 days (13 Sept–13 Nov)	20.12 ± 1.8 h (18.54–05.00)	1.8 ± 1.8 h (0.6–10.3)
All	121	10 Oct ± 15.0 days (13 Sept–13 Nov)	20.11 ± 1.3 h (18.47–05.30)	1.6 ± 1.3 h (0.6–11.7)

**Figure 2.**
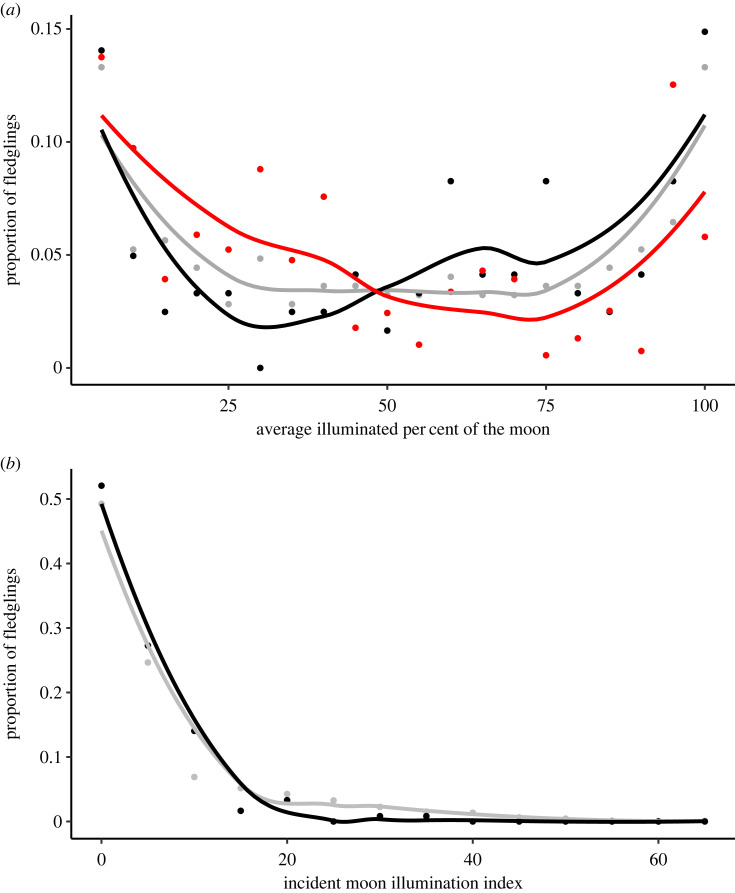
(*a*) Line plot (LOESS line of smoothing) of the observed proportion of chicks that fledged (black) and stranded (red) associated with the nightly average illuminated per cent of the moon (AIPM). These are compared with the expected number of fledglings or stranded birds (grey) should birds strand/fledge randomly relative to AIPM available during the fledging period of 13 September to 13 November in each year. Stranding data (methods and data described in [6], unpublished 2022 data collected using identical methodology by T. V. Burt) were collected from 2019 to 2022 at an illuminated seafood processing plant in Bay de Verde, Newfoundland and Labrador, Canada. Mass stranding events (greater than 100 birds stranded in one night) were excluded from this plot. (*b*) Line plot (LOESS line of smoothing) of the proportion of chicks that fledged (black) at a particular incident moon illumination index (IMII) and the proportion of time available (grey) at each of 5% index intervals during the fledging period (13 September to 13 November) in each year. The IMII is a measure of both moon fullness and its angular position in the sky and did not exceed 65% throughout the fledging period in any year at this location.

Fledging time relative to sunset was similar among years (Kruskall–Wallace *χ*^2^ = 1.31, d.f. = 3, *p* = 0.73). The ANOVA for fledging date was significant (*F* = 2.79, d.f. = 3, *p* = 0.044), though inter-annual pairwise comparisons were not (electronic supplementary material, table S2). AIPM on peak fledge date differed among years (electronic supplementary material, figure S6). During each quarter AIPM, the proportion of chicks fledging when the moon was above or below the horizon did not differ from expected (*χ*^2^ = 0.16, d.f. = 7, *p* > 0.99). The distribution of AIPM on fledging night did not differ from the distribution throughout the fledging season (*D* = 0.12, *p* = 0.19; figure 2*a*, electronic supplementary material, S7 and table S3). The distribution of IMII at the time of fledging differed from the distribution throughout the fledging season (*D* = 0.13, *p* = 0.030; figure 2*b*, electronic supplementary material, S8), where more chicks than expected fledged when IMII was 5–10% (electronic supplementary material, table S4).

## 4. Discussion

Using data from PIT technology, we determined the median fledging date and time of Leach's storm-petrel chicks at Gull Island to be 10 October 1.6 h after sunset (figure 1 and table 1). Fledging ranged from mid-September to mid-November, which aligns with previous reports from colonies in Atlantic Canada [20]. These dates also align with periods of peak strandings reported for Leach's storm-petrels on the island of Newfoundland [5–7,27]. Studies documenting stranded Leach's storm-petrels report that the majority of birds that strand during this period are fledglings [5,6], and it is assumed that these birds stranded on the night they fledged, as observed in other procellariiforms [9,10].

We observed fledging times close to sunset (figure 1 and table 1). While it is unknown for how long storm-petrel fledglings remain at the colony after departing their burrow, these early fledging times concur with findings from surveys of stranded fledgling procellariiforms, which observed peak stranding within a few hours of sunset [9,11]. Future research could verify that stranded storm-petrels are recent fledglings by tracking fledglings during their inaugural flight to investigate the timing and conditions of departure from the colony and determine whether the direction of travel (towards anthropogenically lit areas or out to sea) is influenced by nocturnal illumination (i.e. [9]).

Contrary to our hypothesis, storm-petrels fledged across AIPM, IMII, and regardless of whether the moon was above the horizon (figure 2, electronic supplementary material, S6, S7). This result is surprising for two reasons. Adults tend to reduce their activity at the colony during the full moon [12–15], which may be a predator avoidance strategy, and fewer Leach's storm-petrels strand during the full moon (figure 2*a*) [5,7]. Storm-petrel chicks fledging across moonlight conditions suggests that attraction to anthropogenic light is tempered by available moonlight [19].

Several hypotheses seek to explain why storm-petrels and other nocturnal seabirds are attracted to anthropogenic light. First, seabirds may navigate using moon- and starlight [28], so anthropogenic light may be disorienting and cause them to move towards it. Second, storm-petrels may orient toward anthropogenic light because they mistake it for their bioluminescent prey [29]. Storm-petrels fledging during all moonlight conditions has interesting implications for the navigation hypothesis. If nocturnal seabirds use moonlight to navigate, fewer fledglings may strand during the full moon because increased natural light facilitates navigation. Also, fledglings may be particularly vulnerable to light attraction due to their underdeveloped visual systems [30,31]. Therefore, greater moon illumination reducing the relative intensity of anthropogenic light will presumably reduce their attraction [11,14,19].

Predation avoidance may lead to reduced storm-petrel activity on the colony during a full moon [12]. At the colony, the dominant predators of Leach's storm-petrels are often diurnal charadriiforms such as herring gulls (*Larus argentatus*) and great skuas (*Stercorarius skua*) [21,32,33]. Though these predators can forage at night, they likely benefit from well-lit conditions provided by greater nocturnal illumination [12,34–36]. In response, storm-petrels may avoid detection by remaining inside the burrow or remaining at sea, resulting in low colony activity outside the burrows. This behaviour may be innate as other seabirds have been shown to adjust their activity based on moon phase even in the absence of predation pressure on the colony [37,38]. Some Leach's storm-petrel fledglings, however, depart their burrow under relatively high moon illumination, so subsequent moonlight avoidance behaviour as adults could also be learned. The proportional lack of moonlight avoidance while fledging from Gull Island may be because most gulls are no longer present at the colony when storm-petrel chicks begin to fledge [39,40], so there is little antipredator benefit to chicks to avoid fledging under a full moon.

From a conservation perspective, these results indicate that Leach's storm-petrel fledgling monitoring and rescue programmes should concentrate efforts beginning early in the night throughout mid-September to mid-November. Storm-petrels do not appear to base their fledging decision on moon conditions, however, other factors like wind speed, wind direction, fog, and the brightness and colour of anthropogenic light may influence the likelihood of birds stranding, creating the possibility for mass strandings even during a full moon [4]. Long-term studies of mass-stranding events of all seabird species (i.e. [5,6,8,41,42]) are valuable for determining factors influencing the probability of mass-stranding events. Fledging and stranding information could be used to reduce light pollution during peak fledging periods and high-risk conditions (e.g. foggy conditions during mid-September through November) [43]. Understanding such factors will allow conservation actions to mitigate and respond to stranding events more effectively.

## Data Availability

Fledging data and code are available from the Dryad Digital Repository https://doi.org/10.5061/dryad.2bvq83bws [23]. Supplementary material is available online [44].
